# Prevalence of myocardial hypertrophy in a population of asymptomatic Swedish Maine coon cats

**DOI:** 10.1186/1751-0147-50-22

**Published:** 2008-06-18

**Authors:** Suzanne Gundler, Anna Tidholm, Jens Häggström

**Affiliations:** 1 Bagarmossen Animal Hospital, Ljusnevägen 17, S-128 48 Bagarmossen, Sweden; 2 Albano Animal Hospital, Rinkebyvägen 23, S-182 36 Danderyd, Sweden; 3 Department of Physiology, Faculty of Veterinary Medicine, Swedish University of Agricultural Science, PO box 7018, Uppsala, Sweden

## Abstract

**Background:**

Maine coon cats have a familial disposition for developing hypertrophic cardiomyopathy (HCM) with evidence of an autosomal dominant mode of inheritance [1]. The current mode to diagnose HCM is by use of echocardiography. However, definite reference criteria have not been established. The objective of the study was to determine the prevalence of echocardigraphic changes consistent with hypertrophic cardiomyopathy in Swedish Maine coon cats, and to compare echocardiographic measurements with previously published reference values.

**Methods:**

All cats over the age of 8 months owned by breeders living in Stockholm, listed on the website of the Maine Coon breeders in Sweden by February 2001, were invited to participate in the study. Physical examination and M-mode and 2D echocardiographic examinations were performed in all cats.

**Results:**

Examinations of 42 asymptomatic Maine coon cats (10 males and 32 females) were performed. The age of the cats ranged from 0,7 to 9,3 years with a mean of 4,8 ± 2,3 years. Four cats (9,5%) had a diastolic interventricular septal (IVSd) or left ventricular free wall (LVPWd) thickness exceeding 6,0 mm. In 3 of these cats the hypertrophy was segmental. Two cats (4,8%) had systolic anterior motion (SAM) of the mitral valve without concomitant hypertrophy. Five cats (11,9%) had IVSd or LVPWd exceeding 5,0 mm but less than 6,0 mm.

**Conclusion:**

Depending on the reference values used, the prevalence of HCM in this study varied from 9,5% to 26,2%. Our study suggests that the left ventricular wall thickness of a normal cat is 5,0 mm or less, rather than 6,0 mm, previously used by most cardiologists. Appropriate echocardiographic reference values for Maine coon cats, and diagnostic criteria for HCM need to be further investigated.

## Background

Primary HCM is characterised by concentric myocardial hypertrophy, i.e. increased wall thickness with normal or decreased chamber inner dimensions, primarily of the left ventricle, in the absence of other cardiac, systemic or metabolic abnormalities which may cause myocardial hypertrophy [2,3]. This disease has also been labeled idiopathic HCM, but because some of the underlying causes for HCM have been identified [1,4], primary HCM may be a more appropriate name.

The hypertrophy associated with HCM ranges from localized and mild thickening, involving any part of a particular wall segment, to diffuse and pronounced hypertrophy of the entire chamber [5,6]. With moderate to severe hypertrophy, end-systolic cavity obliteration is often seen at the level of the papillary muscles. As isolated regions of the basal, middle or apical parts of the ventricular walls can be affected, it is necessary that the echocardiographic examination is performed from the right parasternal long-axis view as well as the short-axis view. Because M-mode echocardiograms obtained from the standard view for measuring the left ventricular dimensions, just below the mitral valve leaflets, can miss localized hypertrophy, assessment of ventricular dimensions should be made from both two-dimensional (2D), sweeping from base to apex, and from M-mode [1-3].

There is a wide morphologic spectrum of HCM and no pattern of hypertrophy can be considered typical. This makes the diagnosis of mildly affected cats, often seen in screening programs, difficult. In these cases the cardiologist may have the delicate task to decide if a cat with findings in agreement with less pronounced signs of HCM, like SAM, moderate hypertrophy of the papillary muscles or left ventricular wall of between 5,0 and 6,0 mm, should be considered being affected, and what recommendations should be given to the owner concerning breeding.

Systolic anterior motion (SAM) of the mitral valve is commonly identified in cats with HCM, and can be seen before hypertrophy of the left ventricular wall is evident [1]. SAM is reported to produce dynamic obstruction to left ventricular outflow tract, as well as mitral regurgitation [1,2,5]. Narrowing of the left ventricular outflow tract by hypertrophy of the interventricular septum contributes to dynamic obstruction of the left ventricular outflow tract. Cats with SAM often have a systolic heart murmur, best heard over sternum or left apex [2].

There is evidence suggesting that HCM in certain cat breeds, i.e. American shorthairs [4], Persians [7,8] and Maine coon cats [1] is heritable. A heritable basis for HCM in pigs [9] and pointer dogs [10] is also suspected. Because there is evidence that HCM is heritable in some cats, breeders are increasingly interested in identifying affected cats. Data from a family of Maine coon cats with familial HCM suggest that the disease is inherited in a simple autosomal dominant pattern with complete penetrance.[1].

The aim of the study was to assess the prevalence of myocardial hypertrophy in a population of Maine coon cats.

## Methods

Cats over the age of 8 months, owned by breeders listed on the website of Maine coon cat breeders in Sweden, living in Stockholm, as defined by area code number, by february 2001 were invited to participate in the study. Nine out of ten invited breeders accepted the invitation, and 42 out of 45 possible cats participated in the examinations. Out of these cats, 30 were closely related (mother, father, daughter, son, sister, brother, half sister or half brother) to one or more of the other cats examined in this study. Exclusion criteria consisted of echocardiographic signs of heart disease, other than HCM. None of the invited cats was excluded from the study.

### Clinical examination

Body weight and gender were registered, as was information on whether the cats were castrated or pregnant. Any knowledge of HCM among ancestors was noted. All cats were examined clinically, and any heart murmur auscultated was located and graded on a scale of I-VI [11]. Other causes for concentric left ventricular hypertrophy, such as hyperthyroidism, systemic arterial hypertension or hypersomatotropism were not investigated.

### Echocardiography

Two dimensional (2D) and M-mode echocardiography was performed using an Aloka SSD-2200 with a multifrequence, phased array transducer ranging from 2 to 5 MHz. All echocardiographic examinations were performed by one cardiologist (SG), who was unaware of the cats individual family history at the time of examination. All measurements were made directly on the screen. ECG was simultaneously recorded. The cats were unsedated and gently restrained in right lateral recumbency and the examination was performed from the dependant side of the thorax. Standard right parasternal long-axis and short-axis views were used and echocardiographic examinations analyzed according to the recommendations of the American Society of Echocardiography [12] and the Echocardiographic Committee of the speciality of cardiology, American College of Veterinary Internal Medicine [13]. Measurements of diastolic left ventricular dimensions were made from short axis view on M-mode and in case of local hypertrophy seen on 2D, measurements were made from 2D long axis and short axis view as well. The greatest dimension of these images was used. Presence of papillary muscle hypertrophy, end-systolic cavity obliteration was subjectively evaluated from 2D short axis view, and systolic anterior motion (SAM) of the septal mitral valve leaflet was evaluated from 2D long axis view [1].

Definite diagnosis of primary HCM was based on echoccardiographic demonstration of concentric hypertrophy of the left ventricle, when a region of the left ventricular wall or the entire wall of the left ventricle was ≥ 6 mm thick or when severe papillary muscle hypertrophy was present. Cats with moderate papillary muscle hypertrophy or SAM without hypertrophy of left ventricular wall or the septum were suspected of having the disease [1]. Assessment of the left atrium was made on M-mode as well as on 2D image. When measuring on 2D we used the method later described by Hansson et al [14].

### Statistical methods

All statistical calculations were performed by use of a computerized statistical program [15]. To compensate between differences in body weight between the cats, body surface area (BSA) was used to investigate associations between body size and echocardiographic measurements. Body surface area was calculated according to the formula: BSA (m^2^) = (10,4xBW ^(2/3)^)/100 [16]. Statistical methods included linear regression between body weight and IVSd and LVWd. Outliers were identified using Jacknife distances. Values are given as mean ± 2 standard deviations.

## Results

### Clinical examination

The 42 cats were examined in groups at six different occasions. None of the cats had any history or clinical signs of significant organ or systemic disease. Thirty-two cats were females (eight castrated), and ten were males (eight castrated). One cat was pregnant. The age of the cats varied between 0,7 and 9,3 years (mean 4,8 ± 2,3 years). The body weight for all cats ranged from 3,0 to 8,2 kg (mean 4,9 ± 1,3 kg). The bodyweight of the female cats ranged from 3,0 to 7,0 kg (mean 4,4 ± 0,88 kg) and of the male cats from 4,5 to 8,2 kg (mean 6,5 ± 1,32 kg). One cat had a low grade systolic murmur, II-III/VI, This cat was pregnant (49 days from mating) at the time of initial examination when the murmur was heard. On reexamination, 545 days post partum, no murmur could be detected. None of the examined cats had relatives with confirmed HCM.

### Echocardiographic findings

According to the criteria specified above, 4 cats (9,5%) in the present study were diagnosed with HCM. One of them (a male, 4 years old) had hypertrophy of the entire left ventricular wall, but the basal part of septum was most thickened, (IVSd 9,3 mm, LVFWd 6,4 mm). The papillary muscles were moderatly hypertrophied in this cat, but the left atrium was not dilated. The other 3 cats diagnosed with HCM (1 male, 9 years of age and 2 females, 11 months and 8 years and 4 months old) had regional hypertrophy of the basilar septum (IVSd 7,3; 7,0 and 6,9 mm, respectively) but normal dimensions of the free wall. The male cat in this group, also had moderately hypertrophied papillary muscles.

Two more cats (1 male and 1 female) had wall dimensions < 6,0 mm, but were suspected of having HCM because of SAM. The measurement of the basilar part of interventricular septum of one of these cats was borderline (5,9 mm).

Five cats (2 males and 3 females) had septal dimensions greater than 5,0 mm but less than 6,0 mm. One of them had SAM, as mentioned above, but none had hypertrophy of the papillary muscles. (Table 1). One cat had a free wall of 5,6 mm but septal dimension less than 5,0 mm.

**Table 1:** Data from clinical and echocardiographical examination Age, body weight, mean ± SD and range values for M-mode and 2D echocardiographic parameters in 42 nonsedated, asymtomatic Maine coon cats.

Variable	Mean	SD	Range
Age (Yrs)	4.8	2.3	0.7–9.3
Body weight (kg)	4.9	1.3	3,0–8.2
IVSd (mm)	4.3	1.2	2.3–8.1
LVIDd (mm)	14.8	2.5	8.4–20,0
LVWd (mm)	4.1	0.8	2.9–6.2
IVSs (mm)	6.4	1.6	3.8–12.3
LVIDs (mm)	8.7	2.4	3.0–14.4
LVWs (mm)	6.2	1.4	3.9–10.5
FS (%)	41.8	9.9	25–73
AO-M-mode (mm)	8.9	1.6	6.6–14.3
LA-M-mode (mm)	11.6	2.0	8.2–16.6
LA/AO-M-mode	1.3	0.3	0.8–1.9
AO-2D (mm)	9.0	1.5	6.4–13.1
LA-2D (mm)	11.1	1.6	7.9–15.2
LA/AO-2D	1.3	0.2	0.9–2.2

IVSd = Interventricular septal end-diastolic diameter

LVIDd = Left ventricular inner end-diastolic diameter

LVWd = Left ventricular posterior wall end-diastolic diameter

IVSs = Inter ventricular septal systolic diameter

LVIDs = Left ventricular inner systolic diameter

LVWs = Left ventricular posterior wall systolic diameter

FS = Fractional shortening

AO-M-mode = Aortic end-diastolic diameter measured on M-mode

LA-M-mode = Left atrial end-systolic diameter measured on M-mode

AO-2D = Aortic end-diastolic diameter measured on 2D

LA-2D = Left atrial end-diastolic diameter measured on 2D

Nine cats out of ten with left ventricular wall thickness of > 5,0 mm had asymmetric hypertrophy as defined by IVSd/LVWd ≥ 1,1.

**Figure 1:**
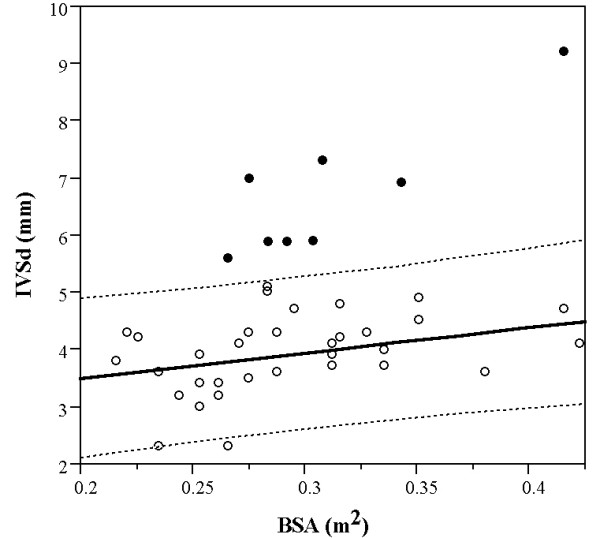
**IVSd correlated to body weight**. Described by outlier analysis in 42 nonsedated asymptomatic Maine coon cats.

The IVSd and LVWd were both significantly correlated to body weight (P < 0,05) with a correlation coefficient of 0,46. Outlier analysis showed that eight cats had IVSd dimensions outside of the Jacknife distance (Figure 1).

## Discussion

Screening programs of apparently healthy animals involve the hazard of over- or underestimating the prevalence of the investigated disease. Therefore, the choice of reference values is a pertinent issue, which merits special attention. There is discrepancy between diagnostic echocardiographic values for HCM used by different cardiologists. Most reports consider an end diastolic thickness at any location of the left ventricular wall of > 6 mm [5,17-19] or ≥ 6 mm [1,5,20] diagnostic of HCM, but reference value of > 5,5 mm [21] and separate values for the interventricular septum of >6 mm and of the free wall of > 5,5 mm [22] is also used.

Assessment of papillary muscle hypertrophy and SAM are subjective, and the inter-operator variability between studies must be considered. It may be expected that the use of screening program will increase the estimated prevalence of HCM. Reexamination of cats with equivocal results is of importance in screening programs.

Echocardiographic reference values for dogs are modified according to body weight, and there are also breed specific references. For adult cats, most cardiologists use reference values irrespective of breed and body size. In Maine coon cats, especially male cats with lean body weights of 8–9 kg, there is reason to question whether reference values should be different from normal sized cats.

A recent report on M-mode measurements in healthy adult Maine coon cats compared to normal domestic cats concludes that mean values of aortic root (Ao), four different systolic measurements (LVIDs, IVSs, LVPWs, LADs) and left ventricular end diastolic diameter (LVIDd) were significantly greater in Maine coon cats compared to domestic cats in general [23]. The diagnostically important dimensions of interventricular septum (IVSd) and left ventricular posterior wall (LVPWd) were however not significantly different from previously reported values for domestic cats. Maine coon cats with echocardiographic parameters consistent with, or suggestive of, a diagnosis of HCM were excluded from the study, but it is not reported what criteria where considered diagnostic. In our study, the cats with abnormal wall thickness were identified using outlier analysis (jackknife distances). We believe that this approach may be more appropriate to establish normal reference range because all cats are included in the initial analysis, and the identification of potentially abnormal cats was not dependant on previously established reference ranges.

No cat in our study had LVIDd above normal reference for domestic cats [5,23]. Mean weight for cats in Drourrs study was 5,5 ± 1,33 kg compared to 4,9 ± 1,3 kg in our study. This difference is probably due to a greater percentage of male cats (44%) compared to our study (24%), which also affects the measurements for all adult Maine coons, male and female together. Using specific reference values according to gender seems appropriate for Maine coon cats.

In HCM the hypertrophy may be symmetrical (septum and free wall equally effected) or asymmetrical (the septum more hypertrophied than the free wall or vice versa) Asymmetrical septal hypertrophy in cats has been defined by one author as septal to free wall thickness ratio ≥ 1,1 [6]. Reports in the literature differ on whether the most common form of HCM among cats is symmetrical or asymmetrical hypertrophy. These differences may to some degree depend on different definitions of asymmetrical septal hypertrophy and methods of measuring the ventricular wall, and determining the ratio between dimension of septum and free wall, but may also be caused by different phenotypic expressions between different populations of cats. In our study, all cats but one who had hypertrophy of the left ventricular wall, had more prominent hypertrophy of septum than of the free wall. That differs from reported findings in a colony of Maine coon cats with familial HCM [1], where Kittleson reported that wall thickening is often localized primarily to the papillary muscles and the posteriolateral free wall, between the papillary muscles. This differs from the human form of familial HCM wich mostly affects the interventricular septum [24], and other reports of feline HCM where asymmetrical septal hypertrophy or symmetric ventricular hypertrophy is the most common form [2,5,19,25-27]. Whether or not this disparity is caused by mutations in different genes, is presently not known.

Our study has a number of limitations. The number of cats and breeders is too small to estimate the prevalence of HCM in the whole population of Swedish Maine coon cats, and reference values for Maine coon cats in general. There is a possibility that cats in Sweden have differences in genotype and phenotype compared to other populations. Because our study was designed as a prebreeding program of supposedly healthy cats, we did not initially investigate other possible causes for concentric myocardial hypertrophy in these cats. However, in February 2007, five years after initial examination, we invited the owners of the four cats with left ventricular wall dimensions of 6 mm or more, to a follow up examination. We then had the opportunity to examine two cats, then 10 and 13 yars old, They had until then not showed any signs of cardiac disease, and we could at the reexamination, by blood samples and measuring of systemic blood pressure, eliminate systemic hypertension, hyperthyroidism and renal failure as causes of myocardial hypertrophy.

The probe used was a multifrequence probe 2–5 MHz. A probe with higher frequency would have given more detailed information that to some degree may have influenced the outcome of the examinations.

A genetic test for a specific mutation in the myosin binding protein C gene, found among Maine coon cats with familial HCM, is now available [28,29], which has led to cats being genetically screened. The value of this test in Maine coon cats outside that colony and in the general cat population has not yet been evaluated. There are indications that other genes may cause HCM in cats, which indicates that echocardiographic screening of the general cat population is still important.

## Conclusion

Irrespective of the reference values used, the prevalence of echocardiographic findings consistent with HCM in this population of asymptomatic cats was high.

Using a reference value of ≥ 6 mm for the left ventricular wall, we found that in the present study 4 (9,5%) cats were definitively diagnosed with HCM, and two additional cats with SAM were suspected of having the disease, which added together makes 14,3% of this population. The most interesting result of our study is that it suggests that the left ventricular wall thickness of a normal cat is 5,0 mm or less. Using this value as reference, 5 (11,9%) more cats with a left ventricular wall thickness of more than 5,0 mm but less than 6,0 mm can be suspected of being affected with HCM. In that case a total of 11 (26,2%) cats will be considered abnormal.

By echocardiography, we are however unable to detect cats that carry mutant genes, but have mild or no echocardiographic changes. Hopefully, it will in the future be possible to identify all specific mutations causing HCM carriers by analysis of blood samples, but until then more studies about normal reference values and diagnostic criteria for HCM are needed.

## Competing interests

The authors declare that they have no competing interests.

## Authors' contributions

SG participated in the design of the study, carried out the clinical and echocardiographical examinations and drafted the manuscript.

AT participated in the design of the study, participated in the examinations of the cats and helped to revise the manuscript.

JH participated in the design of the study, performed the statistical analysis and interpretation of data and helped to revise the manuscript.
